# The spectrum of cutaneous polyarteritis nodosa. A case report of two contrasting cases and review of the literature

**DOI:** 10.1002/ccr3.6659

**Published:** 2022-11-27

**Authors:** Ian Liang, Mariya Hamid, Deshan Sebaratnam, Nicholas Manolios

**Affiliations:** ^1^ Department of Rheumatology Westmead Hospital Sydney New South Wales Australia; ^2^ Faculty of Medicine and Health The University of Sydney Sydney New South Wales Australia; ^3^ Department of Dermatology Liverpool Hospital Sydney New South Wales Australia; ^4^ Faculty of Medicine University of New South Wales Sydney New South Wales Australia

**Keywords:** cutaneous, medium vessel, Polyarteritis nodosa, polyarteritis nodosa, vasculitis, vasculitis case report

## Abstract

Cutaneous polyarteritis nodosa is a rare neutrophilic vasculitis. We present two cases that reflect the gamut of this disorder including one case whose delayed diagnosis led to permanent nerve deficit and scarring.

## Graphical abstract

Cutaneous polyarteritis nodosa is a distinct entity different from polyarteritis nodosa that requires prompt recognition and treatment to prevent permanent neurological deficit and scarring.

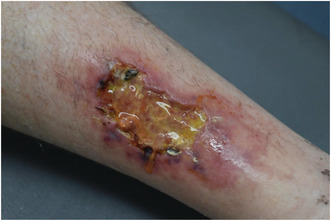

## 1 INTRODUCTION

cPAN is a rare form of vasculitis that involves the small‐ to medium‐sized arteries and has a more benign course and a more favorable prognosis compared to systemic PAN.^1^ Due to the rarity of this condition, the current evidence‐based management for this condition has only been limited to case reports and case series with no randomized controlled trials to inform the best approach to management. These two cases highlight the spectrum of cPAN as well as one approach to its management.

## 2 CASE PRESENTATION

### 2.1 Case 1

**FIGURE 1:**
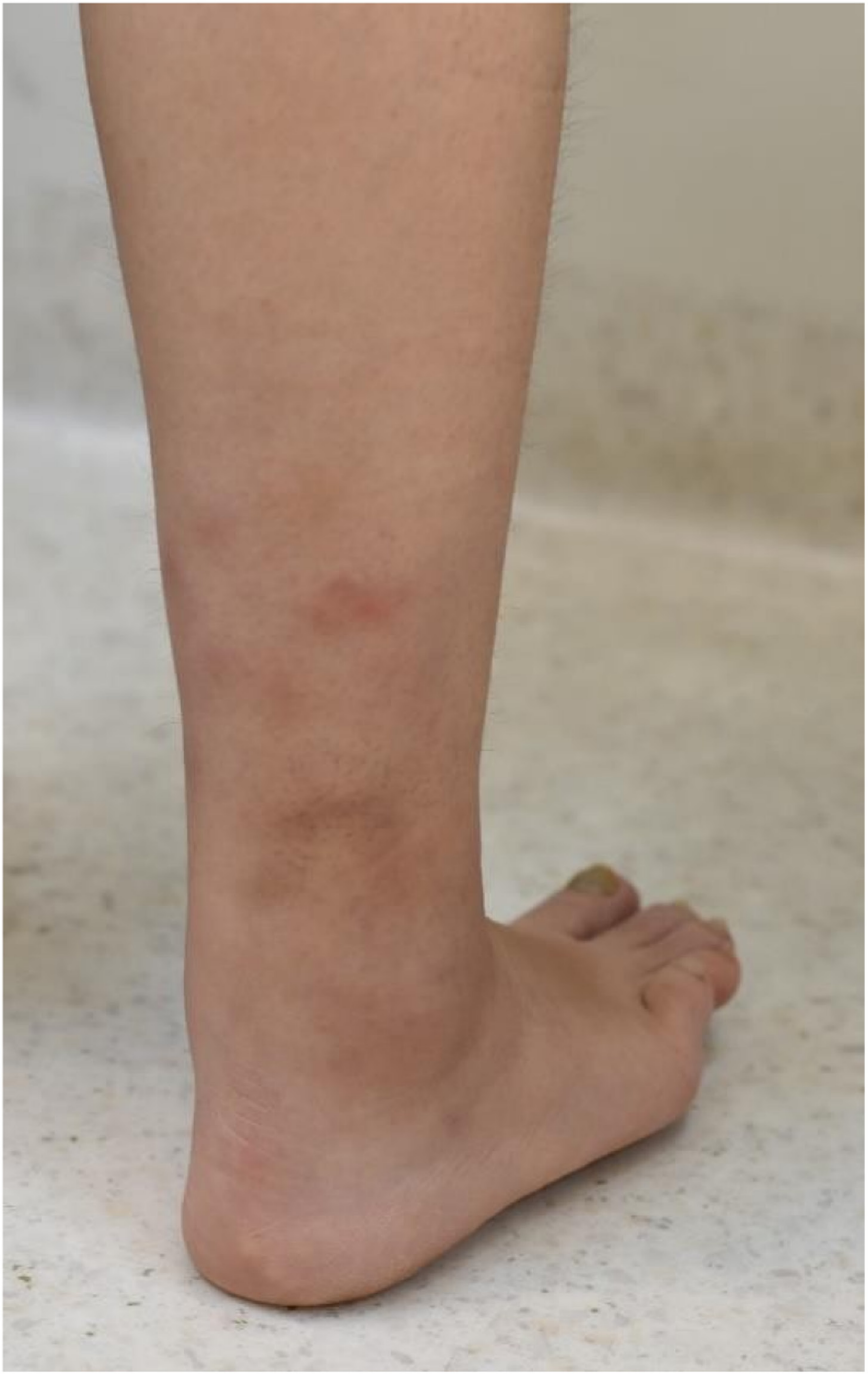
Erythematous subcutaneous nodules and angular purpura on the posterior lower legs

**FIGURE 2:**
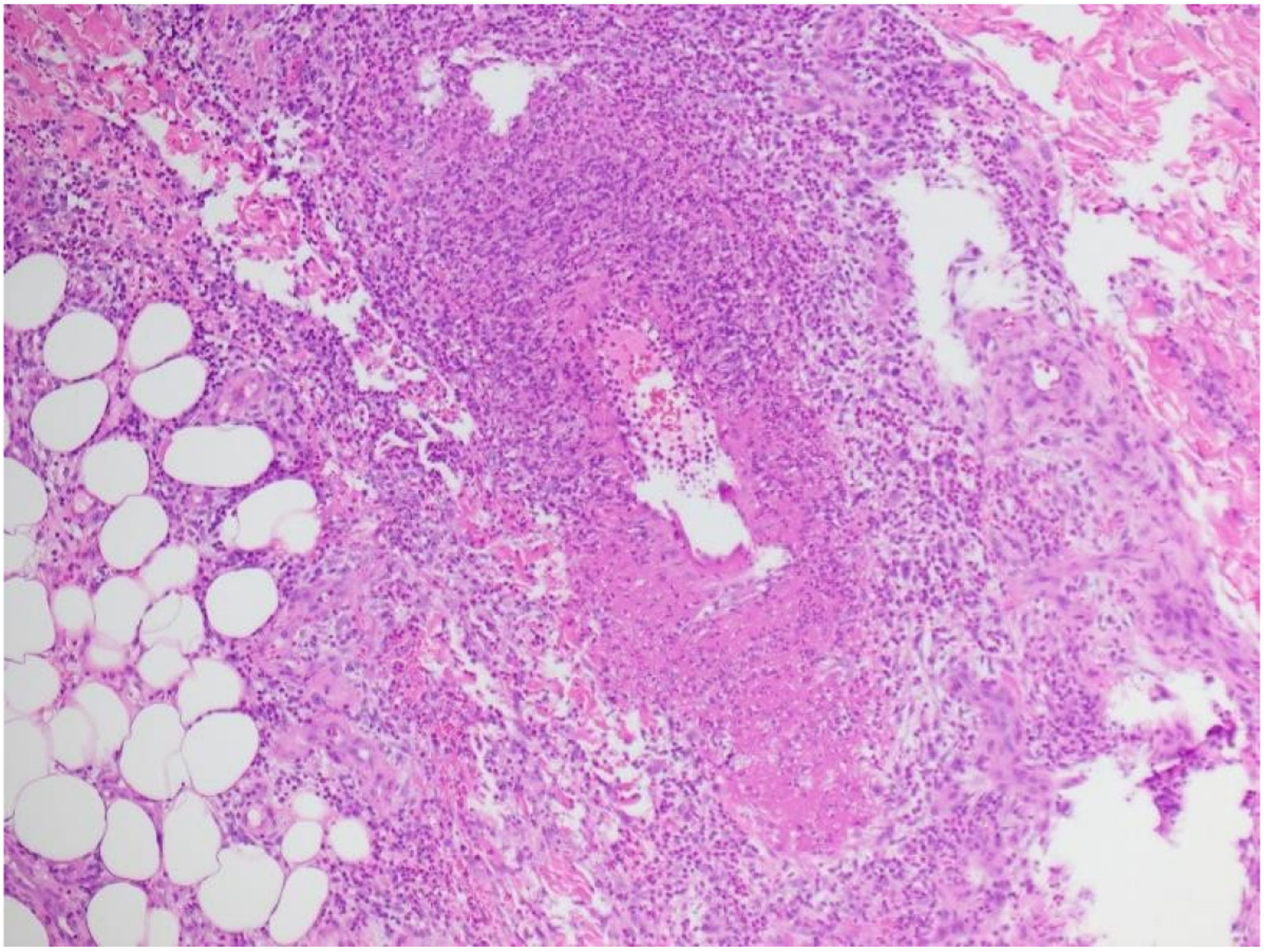
Histopathological section of lower limb subcutaneous nodule. The findings demonstrated a mixture of lymphocytes, neutrophils, histiocytes, and scatted eosinophils surrounding the small arteries and extending into the fibrous septa and peri‐septal lobules of the subcutaneous tissue. The internal elastic lamina of the arteriole was preserved. There were no granulomas, and further stains indicated no mycobacterial or fungal elements. These findings were consistent with PAN.

A 34‐year‐old Filipino female presented with a three‐month history of multiple bilateral tender red nodules over the distal lower limbs (Figure 1). She felt well with no constitutional symptoms, no prior history of preceding infection, tuberculosis exposure, or animal contact. She had migrated from the Philippines 5 years ago. She had no smoking, alcohol, or intravenous drug use. Prior to this episode, she had no significant medical issues and was not on any medications or herbal supplements. On examination, she had tender erythematous subcutaneous nodules on her lower limbs with angular purpura (Figure 1). There were bilateral ankle effusions and no other synovitis elsewhere. The abdomen was soft and non‐tender. Urinalysis, full blood count, biochemistry, and creatinine kinase levels were normal. Erythrocyte sedimentation rate (ESR) was 44 mm/hr (N < 20 mm/h) and C‐reactive protein (CRP) 10 mg/dl (N < 4 mg/dl). Antinuclear antibody, extractable nuclear antigen, cryoglobulin, cryofibrinogen, rheumatoid factor, anticardiolipin antibody, β‐2 glycoprotein antibody, lupus anticoagulant, antineutrophil cytoplasmic antibodies, hepatitis B, hepatitis C, HIV, measles, mumps, and rubella were negative. The interferon‐gamma release assay was negative. Incisional biopsy demonstrated fibrinoid necrosis of an arteriole with a marked perivascular infiltrate composed of lymphocytes, neutrophils, histiocytes, and eosinophils, extending to the subcutis. Histiocytes were inconspicuous, and PUTT and ABPAS staining demonstrates no pathogens. (Figure 2). Whole‐body PET‐CT and CT abdominal angiogram showed no systemic vessel involvement or end‐organ ischemia. Prednisone 40 mg daily was commenced with normalization of ESR, CRP, and complete resolution of subcutaneous nodules after 2 weeks. Prednisone was reduced and methotrexate commenced as a steroid‐sparing agent.

### 2.2 Case 2

**FIGURE 3:**
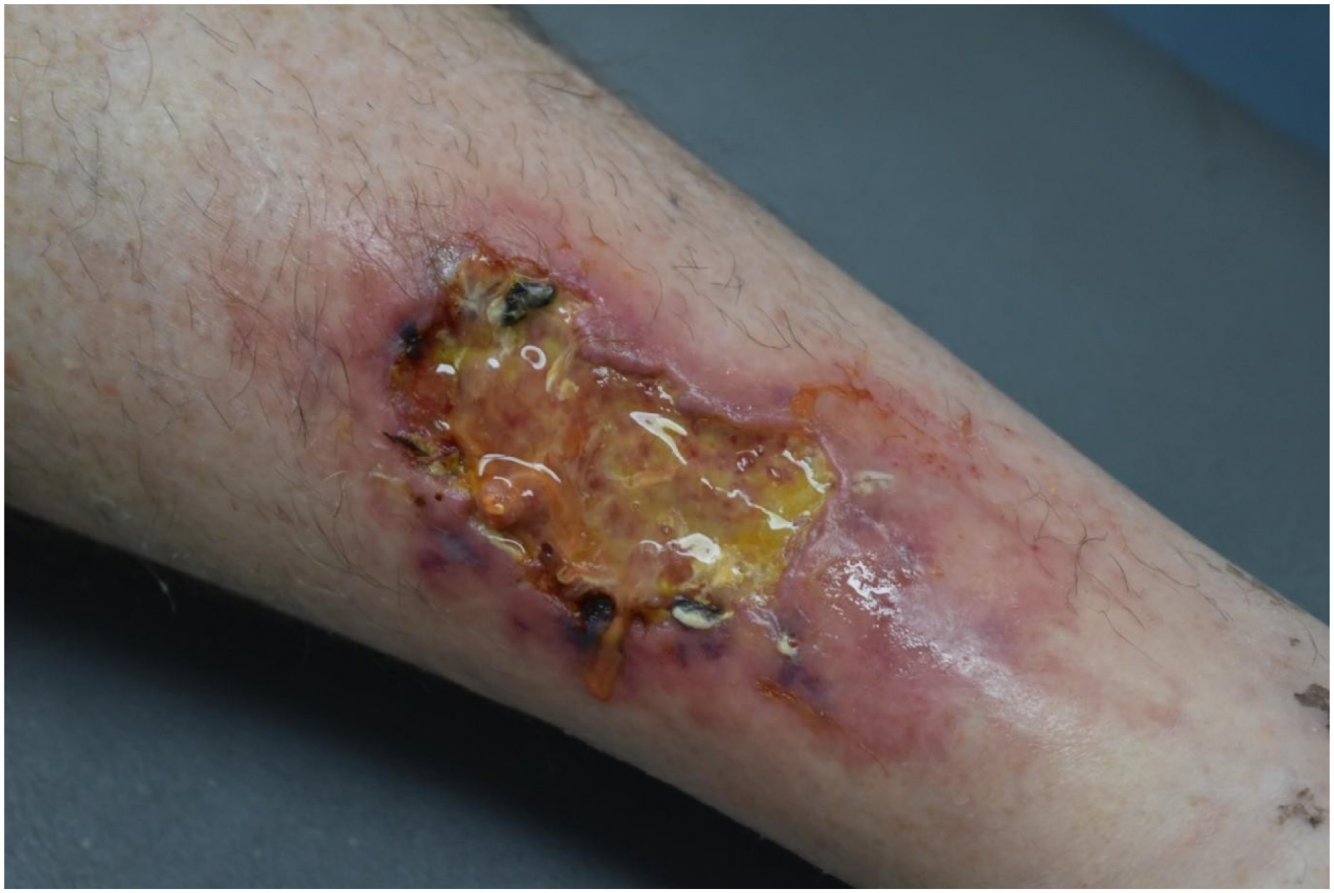
Stellate ulcer with retiform purpura. The incisional biopsy demonstrates a prominent lymphohistiocytic infiltrate of small‐ to medium‐sized vessels in the deep dermis and subcutis. A moderate lymphohistiocytic infiltrate was noted within vessel walls with endarteritis obliterans, degenerate vessel walls, and fibrin thrombi with karyorrhexis in the affected lumen.

A 55‐year‐old man was referred with a five‐month history of right lower limb ulcer associated with sensory changes in the lower limb. There were no constitutional symptoms or other symptoms to suggest systemic disease. Examination demonstrated a stellate ulcer with retiform purpura (Figure 3) and sensory deficit along the sural nerve. Physical examination was otherwise unremarkable. An incisional biopsy demonstrated a prominent lymphohistiocytic infiltrate of small‐ to medium‐sized vessels in the deep dermis and subcutis. A moderate lymphohistiocytic infiltrate was noted within vessel walls with endarteritis obliterans, degenerate vessel walls, and fibrin thrombi with karyorrhexis in affected lumens. His investigations showed normal urinalysis, blood count, urea, and electrolytes with no features of systemic involvement on imaging. Nerve conduction studies demonstrated isolated right common peroneal neuropathy with no evidence of a generalized neuropathy or mononeuritis multiplex. The patient was commenced on a tapering regime of prednisone 60 mg daily, methotrexate 15 mg weekly, and colchicine 0.5 mg tds with resolution of the ulcer over a period of months. The sensory deficit remained fixed. Follow‐up 1 year later showed no recurrence of symptoms or signs of vasculitis.

## 3 DISCUSSION

cPAN can affect all ages with the average age of diagnosis being 40–50 years old with a greater predominance in women.^2^ The estimated prevalence is 31 cases in 1 million, and it is estimated that it accounts for 4% of all cases of polyarteritis nodosa.^3^ In contrast to PAN, cPAN is limited to the skin and associated muscles and joints surrounding the affected area.^2^ Clinical signs include tender subcutaneous nodules typically affecting the lower limbs, which may progress to ulceration in 50% of the cases.^1^ Other dermatological features include livedo reticularis, livedo racemosa, and purpura.^1^ Late‐stage features are necrosis, neuropathy, myositis, and neuritis.^1^ cPAN can be distinguished from sPAN and small vessel vasculitis such as granulomatous polyangiitis by its lack of systemic involvement, cranial nerve palsies, hypertension, bullae, livedo reticularis gangrene of extremities, leucocytosis, and eosinophilia.^2^, ^4^, ^5^ Unlike PAN, it is not fatal if left untreated but follows a benign course that may either become chronic or relapsing and remitting over several years.^1^

Multiple associations with cPAN include group A beta‐hemolytic streptococcus infections, hepatitis B and C, inflammatory bowel disease, CMV, parvovirus B19, tuberculosis, malaria, and drugs such as minocycline, sulfonamides, and IV amphetamines.^1^, ^2^ An incisional biopsy to deep fat for histopathology is mandatory to establish the diagnosis, as well as tissue biopsy for microbial culture depending on the clinical context. Differential diagnoses for other forms of small to medium‐vessel vasculitis include lymphocytic thrombophilic arteritis, panniculitis, venous thrombosis, and atypical infection. Further investigations should include cryoglobulins, ANA, ANCA, RF, and complement levels to exclude secondary causes^1^, ^2^, ^6^ and imaging to exclude systemic organ involvement.

There is no consensus on initial treatment, dosage, length of treatment, or specific drug combination.^7^, ^8^ Current studies have noted that mild cPAN can be adequately treated with NSAIDs, colchicine, topical corticosteroids, and low‐dose corticosteroids.^1^, ^6^, ^8^ The presence of ulceration in the initial episode predicts an increased risk of relapse and is associated with a worse prognosis.^9^ In such patients, case series have demonstrated up to 1 mg/kg/day of prednisone was associated with a significant reduction of pain, disappearance of subcutaneous nodules, and significant improvement of cutaneous ulcers.^1^, ^10^ For patients who were refractory to prednisone, the addition of IV cyclophosphamide, azathioprine, and IVIG has been shown to induce remission.^5^, ^8^, ^11^ Studies have also advised for aggressive treatment for patients who had constitutional symptoms, severe course of disease, or high acute phase reactants.^7^, ^8^ Studies have found that a flare of cPAN usually follows attempts to wean prednisone.^10^ Steroid‐sparing agents include colchicine, hydroxychloroquine, methotrexate, sulphapyridine, pentoxifylline, and dapsone.^1^, ^6^, ^7^ Patients with cPAN should be followed up twice yearly and have regular surveillance to exclude possible progress to systemic PAN.^1^ The likelihood of cPAN progressing to sPAN is rare. In a large case series examining 79 patients with cPAN, there was no progression to systemic PAN in a follow‐up period of up to 30 years.^10^ However, a case series noted that patients have developed sPAN up to 19 years after the initial diagnosis.^12^

## AUTHOR CONTRIBUTIONS

**Ian Liang:** Data curation; formal analysis; investigation; writing – original draft. **Mariya Hamid:** Conceptualization; formal analysis; supervision; writing – review and editing. **Deshan Sebaratnam:** Data curation; supervision; writing – original draft; writing – review and editing. **Nick Manolios:** Conceptualization; data curation; investigation; methodology; project administration; supervision; validation; writing – review and editing.

## CONFLICTS OF INTEREST

On behalf of all authors, the corresponding author states that there is no conflict of interest.

## CONSENT TO PARTICIPATE

Informed consent was obtained from all individuals included in the case report.

## CONSENT FOR PUBLICATION

The patients featured within this case report have provided written consent to grant to any third party, in advance and in perpetuity, the right to use, reproduce, or disseminate the article in its entirety or in part, in any format or medium.

## Data Availability

The authors confirm that the data supporting the findings of this study are available within the article (and/or) its supplementary material.
